# Study on the effects of different pectinase/cellulase ratios and pretreatment times on the preparation of nanocellulose by ultrasound-assisted bio-enzyme heat treatment

**DOI:** 10.1039/d2ra08172e

**Published:** 2023-02-09

**Authors:** Xiaoxiao Wu, Xushuo Yuan, Jiaxin Zhao, Decai Ji, Haiyang Guo, Wentao Yao, Xiaoping Li, Lianpeng Zhang

**Affiliations:** a Yunnan Provincial Key Laboratory of Wood Adhesives and Glued Products, Southwest Forestry University Kunming 650224 Yunnan China lxp810525@163.com lpz@zju.edu.cn; b College of Biological, Chemical Sciences and Engineering, Jiaxing University Jiaxing 314001 Zhejiang China guohy@zjxu.edu.cn

## Abstract

With the development of science and technology, efficient, fast and green methods are increasingly being pursued. The production of nanocellulose by green methods, such as bio-enzymes-assisted ultrasound treatment, has been the focus of many studies. However, the yield of cellulose nanocrystals prepared by this method is very low. In this paper, by pretreatment of microcrystalline cellulose (MCC), nanocellulose was prepared by heating and stirring + pectinase/cellulase + ultrasonic treatment (HSt – P/C – Ultr). The effects of the ratios of pectinase and cellulase and the hydrolysis time on the yield of nanocellulose were studied. FTIR, XRD, SEM, TEM and TG were used to determine the structure, crystallinity, morphology and thermal stability of nanocellulose. The results showed that optimal hydrolysis conditions were determined as a pectinase : cellulase ratio of 1 : 1, 90 min and 50 °C. The yield of nanocellulose was about 32.0%. The yield of pectinase cellulase = 1 : 1 was higher than that of microcrystalline cellulose (MCC) treated by a single bio-enzyme. This indicated that the synergistic effects of pectinase and cellulase have a certain effect on the formation of nanocellulose. During the preparation, the crystalline form of cellulose did not change. It was still cellulose I with a crystallinity of 73.5%, which is 9.50% higher than that of microcrystalline cellulose (MCC), a width of 20–50 nm, a high aspect ratio and a winding network structure. Therefore, nanocellulose prepared by this method is an ideal toughening material for manufacturing composite materials.

## Abstract 2

With the development of science and technology, efficient, fast and green methods are increasingly being pursued.

## Graphical abstract

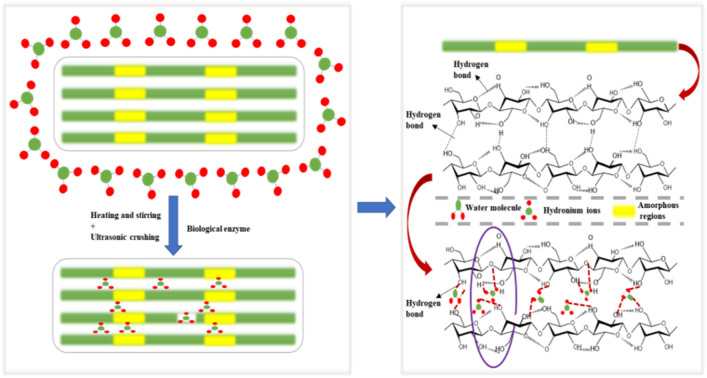

## 1 Introduction

With progress in science and technology, the concept of green environmental protection has been gradually enhanced and the green and efficient preparation of nanocellulose has attracted significant attention from researchers.^1,2^ This is because nanocellulose is a new type of nanomaterial with a one-dimensional size of about 1 to 100 nm,^3^ and compared with natural cellulose, nanocellulose has many unique properties, including natural renewability, biodegradability, biocompatibility, high strength factor, high specific surface area, high aspect ratio, mechanical flexibility and thermal stability.^4–6^ Therefore, the application of nanocellulose in composite materials is of great importance. There are generally three types of nanocellulose: ① cellulose nanofibers, which are mainly prepared by chemical-physical methods, with an average size of 5–60 nm and a length of several micrometers.^7^

② Cellulose nanocrystals are mostly prepared by acid to obtain hydrolyzed fibers with an average size of 5–70 nm and length of 100–250 nm.^8^ ③ Bacterial nanocellulose is synthesized using bacteria; it has an average size of 2–100 nm wide and is about 100 nm long.^9^

In this study, nanocellulose (CNF) was prepared by processing MCC. The existing nanocellulose preparation mainly includes chemical, physical and biological methods. Although acid hydrolysis for the preparation of nanocellulose is efficient, it also has high equipment requirements.^10^ It causes environmental pollution and the formed cellulose nanocrystals have reduced thermal stability.^11–13^ Acid hydrolysis involves the formation and dissociation of hydrogen bonds.^14^ Although the physical method of preparing nanocellulose will not yield products that are unfriendly to the environment, the efficiency of the physical method is very low.^15^ The most typical biological method involves bio-enzymes. Bio-enzyme hydrolysis is green and environmentally friendly and does not produce hazardous waste.^16^ It involves decomposition by bio-enzymes and yields a polydisperse product.^17–19^ According to the literature, there are several methods for preparing nanocellulose with bio-enzymes, which are used either alone or in combination with chemical or mechanical methods.^20,21^ In most cases, this is related to a decrease in the particle size, porosity, or crystallinity of cellulose.^22^ For the selection of pretreatment conditions, researchers prefer efficient, fast and green methods. Because of the green and environment-friendly characteristics of bio-enzymes, they have attracted significant attention from researchers.^23^ Marta Babicka *et al.* obtained cellulose from bio-enzymes-pretreated Avicel cellulose by hydrolysis with EmimOAc, with a particle size of about 200 nm.^24^ Yue *et al.* prepared nanocellulose by the pretreatment of poplar using steam blasting, followed by bio-enzyme-assisted ultrasonic treatment^25^ using only cellulase, and the yield of nanocellulose was about 13.2%. The preparation of nanocellulose by ultrasonic treatment assisted by bio-enzymes gives a low yield and therefore, it is usually combined with other methods and centered on the bio-enzymes themselves.^26^ To overcome the shortcomings of single bio-enzymes, compound bio-enzymes have been used for synergistic action and play better roles in bio-enzyme catalysis. In addition, the method of heating and stirring was selected for the treatment of raw materials to further accelerate the action of bio-enzymes. Ultrasonic treatment facilitates the formation of nanocellulose, and bio-enzyme-assisted ultrasonic treatment is a high-efficiency method.^27^ Given the advantages of both biological and mechanical methods, bio-enzyme-assisted sonication pre-treatment is a promising method for the preparation of CNF. Therefore, heating and stirring + bio-enzymes + ultrasonic treatment constitute a fast and green method. However, there are still some problems of low efficiency in the preparation of nanocellulose by bio-enzyme-assisted ultrasonic treatment, which need to be further explored to find a way to compensate for the efficiency problem.

**Fig. 1:**
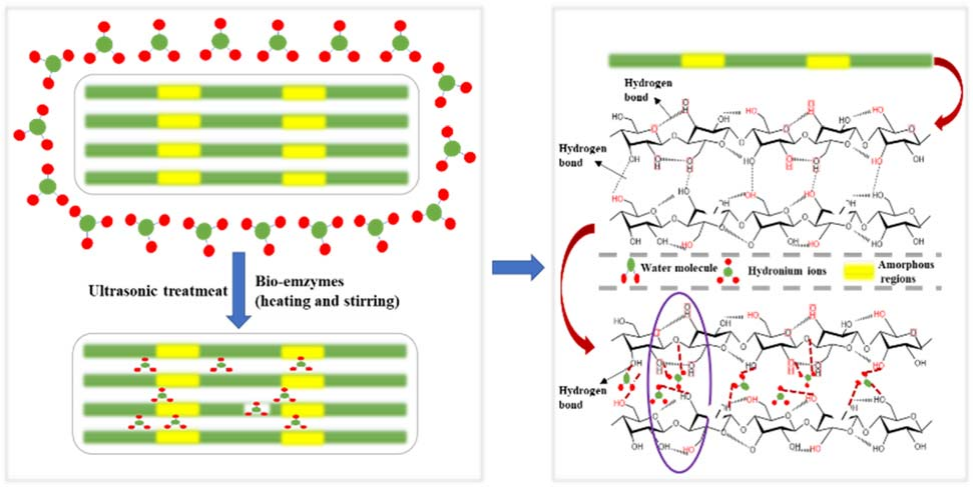
Schematic diagram of nanocellulose preparation.

Herein, to improve the yield of CNC prepared by bio-enzyme-assisted ultrasonic treatment, a new method has been developed. MCC was pretreated with heating and stirring + bio-enzymes + ultrasonic treatment. Pectinase and cellulase were used to make up for the low efficiency of a single bio-enzyme treatment. The synergistic effects of pectinase and cellulase accelerated the formation of nanocellulose; the cellulose-1,4 glycosidic bond was broken, leading to the efficient preparation of nanocellulose. Fig. 1 shows the principle of bio-enzyme pretreatment; the main reasons for the formation of nanocellulose are hydrogen bond breakage and hydrogen bond formation. Bio-enzyme hydrolysis can be promoted by heating and stirring but thermal treatment alone is not enough. Additional bio-enzyme-assisted ultrasonic treatment was performed to prepare nanocellulose, and two-stage bio-enzyme-assisted ultrasonic thermal treatment was performed to further improve the nanocellulose yield. Different ratios of bio-enzymes and different pretreatment times were investigated in this process. The nanomaterials were characterized by Fourier transform infrared spectroscopy (FTIR), X-ray diffraction (XRD), scanning electron microscopy (SEM), transmission electron microscopy (TEM) and thermogravimetry (TG). The structure, surface functional groups, crystal morphology, crystallinity, microscopic characteristics and thermal stability of nanomaterials were analyzed. Exploring the best conditions for the preparation of nanocellulose MCC by bio-enzyme treatment is in line with the concept of environmental protection, which is of great significance for the application of nanocellulose in composite materials.

## 2 Experimental

**Fig. 2:**
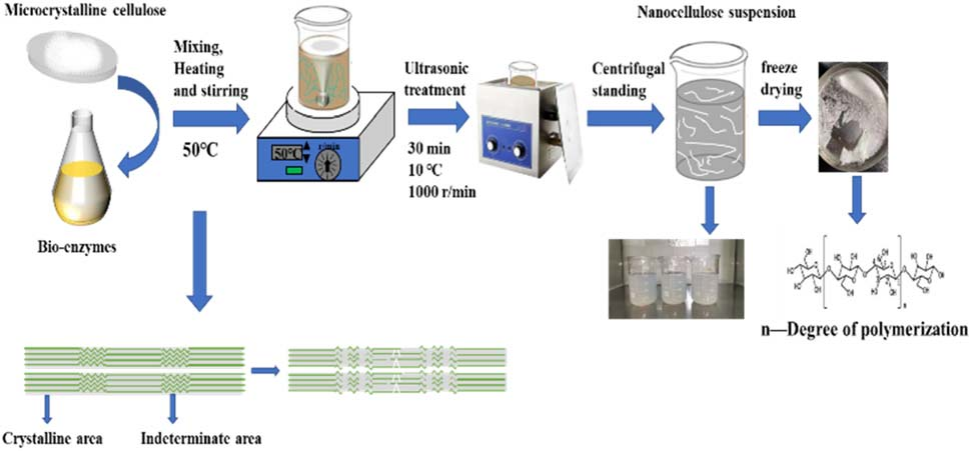
A schematic representation of the experimental process.

A schematic representation of the experimental process is shown below in Fig. 2.

### 2.1 Materials and instrumentation

#### 2.1.1 Materials

Microcrystalline cellulose (Q/CYDZ 2320-2009), was obtained from Sinopharm Chemical Reagent Co. Zirconia grinding beads (0.1 mm) were purchased from China Europe New Materials Co. Cellulase (CEL-01) was purchased from Cangzhou Xia Sheng Enzyme Biotechnology Co. Ltd., with cellulase activity of 30 000 U g^−1^. Pectinase was purchased from Aladdin Biotechnology Co. Ltd. (Shanghai, China). The decomposition activity of pectinase was 30 000 U g^−1^. All reagents were analytically pure.

#### 2.1.2 Instrumentation

Magnetic Heating Stirrer (DF-101Z, Hangzhou Aipu Instrument Equipment Co., Ltd.). Electric Heating Blast Dryer (DHG-9003, Shanghai Yiheng Scientific Instruments Co., Ltd.). Electronic balance (BSM220, Shanghai Zhuojing Electronic Technology Co., Ltd.). Constant-temperature water bath (B220, Shanghai Yarong Biochemical Instrument Factory). CNC ultrasonic cleaner (KQ-250DB, 40 kHz, 250 W, Kunshan Ultrasonic Instrument Co. Ltd., China). Electric centrifuge (Model 80-1, Shanghai Meixiang Instrument Co., Ltd., China). Freeze dryer (SCIENTZ-10N type, Ningbo Xinzhi Biological Co., Ltd., China). Infrared spectrometer (IS50, Seaside Fiserv). X-ray diffractometer (LabX6000, Shimadzu, Japan). Scanning electron microscope (SEM) (Zeiss Sigma300, Sci-go Instruments test platform). Transmission electron microscope (TEM) (Jem-2100F, Sci-go Instruments test platform). Thermogravimetric analyzer (PerkinElmer, USA).

### 2.2 Experimental variable factors

The ratios were set as follows. Pectinase : cellulase = 1 : 0, pectinase : cellulase = 0 : 1, pectinase : cellulase = 1 : 1, pectinase : cellulase = 3 : 7, pectinase : cellulase = 7 : 3 and pectinase : cellulase = 1 : 1. When the ratio of bio-enzymes was pectinase : cellulase = 1 : 1, the pretreatment times for the bio-enzymes were set as 30 min, 60 min, 90 min, 120 min and 150 min.

### 2.3 Preparation of nanocellulose by the HSt – P/C – Ultr method

#### 2.3.1 Bio-enzyme pretreatment stage

Nanocellulose was prepared by pretreatment with bio-enzymes.^28^ First, an aqueous solution containing 5% (mass ratio) cellulase and 5% (mass ratio) pectinase was prepared. About 0.5 g MCC was placed in a beaker containing 20 g zirconia grinding beads (0.1 mm diameter) and a magnetic stirrer. Next, 30 mL of the above-mentioned aqueous solution of pectinase and cellulase in certain volume ratios (1 : 0, 0 : 1, 1 : 1, 3 : 7, 7 : 3) was added to the beaker. The mixed solution was stirred for 30 min, 60 min, 90 min, 120 min, 150 min using a collector-type thermostatic magnetic stirrer (DF-101Z) at 50 °C and 800 rpm.

#### 2.3.2 Ultrasound-assisted heating stage

The mixtures pretreated with the bio-enzymes were placed in a 100 °C water bath for 30 min and then subjected to ultrasonic treatment for 30 min to inactivate the bio-enzymes. The mixture was then centrifuged at 8000 rpm for 10 min and the supernatant fluid was poured off. Next, the sediment was washed with a suitable amount of distilled water to remove the enzyme impurities. This was repeated several times, and then the sediment was collected and diluted with distilled water. The mixtures were again stirred at room temperature for 30 min, ultrasonicated for 30 min, and centrifuged for 10 min. After more than 30 repetitions, centrifugation was performed and the cloudy upper layer was collected and left to stand for about two weeks at room temperature without stratification. This suspension contains the nanocellulose. Finally, the suspension was dried in a freeze dryer (SCIENTZ-10N, Ningbo Xinzhi Biological Co. Ltd., China) and nanocellulose solid was obtained for characterization.

### 2.4 Analysis and characterization

#### 2.4.1 Calculation of nanocellulose yields

The nanocellulose suspension was dispersed uniformly, the total volume was determined, 100 mL was measured in a weighing bottle, vacuum freeze-dried until the mass was constant, and the yield (%) of nanocellulose was calculated using the following eqn (1):^29^1
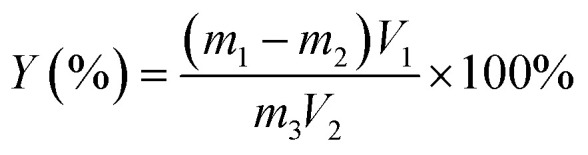
where *Y* is the yield of nanocellulose, %; *m*_1_ is the total mass of the dried sample and weighing bag, g; *m*_2_ is the mass of the weighing bag, g; *m*_3_ is the mass of microcrystalline cellulose, g; *V*_1_ is the total volume of nanocellulose suspension, mL; *V*_2_ is the volume of nanocellulose suspension used for drying.^30^

#### 2.4.2 Fourier transform infrared spectroscopy (FTIR) analysis

FTIR spectroscopy was used to characterize the obtained material and to determine its chemical structure. All samples (1 mg) were mixed with KBr (300 mg) and pressed into tablets for analysis. The spectra were recorded in the range of 4000–400 cm^−1^ with a resolution of 4 cm^−1^.

#### 2.4.3 X-ray diffraction (XRD) analysis

The molecular structure of cellulose was hydrolyzed with different pre-treatment times of bio-enzymes. XRD patterns were obtained to study the changes in the structure, as well as the crystallinity of the nanocellulose prepared under different conditions. Cellulose has a crystalline structure. In the crystalline region, microfibrils are arranged in an orderly manner and they are connected by hydrogen bonds. This fraction of cellulose is highly recalcitrant to bio-enzymes and other chemicals. Some studies have pointed out that fiber crystallinity affects the rate of conversion of bio-enzymes, especially the rate of the initial bio-enzyme hydrolysis.^23^ The diffractometer was equipped with a Cu-Ka emission source, with a test range of 5° to 60° and a test speed of 0.02° s^−1^. The relative crystallinity (crystalline index) of nanocellulose was calculated according to eqn (2):^31^2
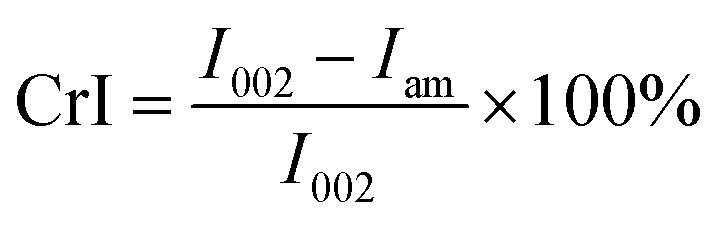
where *I*_002_ is the diffraction peak of the main peak around 22°, and *I*_am_ is the diffraction intensity of the amorphous region around 15°.^32^

#### 2.4.4 Scanning electron microscope (SEM) analysis

The surface morphology of nanocrystalline cellulose was examined using the SEM method, where the degree of nanocellulose refinement could be observed. The average diameter of nanocellulose, which is generally <100 nm, was calculated using Image J software (National Institutes of Health, USA) and Origin software (OriginLab Company, USA).

#### 2.4.5 Transmission electron microscopy (TEM) analysis

The microscopic morphology of nanocellulose was characterized using transmission electron microscopy, and the homogeneously dispersed nanocellulose suspensions were placed on a copper mesh for sample preparation. The samples were dried at room temperature before analysis, with an operating voltage of 80 kV.^33^

#### 2.4.6 Thermogravimetric (TG) analysis

The thermal stability of nanocellulose was analyzed using a thermogravimetric analyzer (NETZSCH STA 2500A-0048-N, 30 °C/10.0 (K min^−1^) 600 °C, China). The mass loss of nanocellulose at different stages was observed to determine the rate of thermal decomposition of cellulose.

## 3 Results and analysis

### 3.1 The effects of different bio-enzyme ratios on the preparation of nanocellulose

#### 3.1.1 Analysis of the yield of nanocellulose

The preparation of nanocellulose by pretreatment of MCC is shown in Table 1. The yield from pectinase treatment reached 30.0%, the yield from cellulase reached 27.0%, the yields from pectinase : cellulase = 3 : 7 and pectinase : cellulase = 7 : 3 were 24.0% and 28.8%, and the yield from pectinase : cellulase = 1 : 1 was 32.0% after 90 min of treatment. The yield from pectinase : cellulase = 1 : 1 was greater as compared to the other four groups. This shows that when the pretreatment time is fixed, the synergistic effects of pectinase and cellulase will further accelerate the cleavage of cellulose-1,4 bonds and the effect is greater than that of a single bio-enzyme, so the yield of nanocellulose is relatively high.

**Table 1:** Calculation of the yield of nanocellulose^a^

Sample category		*m*_1_ (g)	*m*_2_ (g)	*m* (g)	*V*_1_ (mL)	*V*_2_ (mL)	*Y* (%)
90 min	P : C = 1 : 0	2.22	2.19	0.50	500.0	100.0	30.0
	P : C = 0 : 1	2.27	2.24	0.50	450.0	100.0	27.0
	P : C = 1 : 1	2.32	2.28	0.50	400.0	100.0	32.0
	P : C = 3 : 7	2.29	2.26	0.50	400.0	100.0	24.0
	P : C = 7 : 3	2.26	2.23	0.50	480.0	100.0	28.8
P : C. = 1 : 1	30 min	2.29	2.27	0.50	500.0	100.0	20.0
	60 min	2.23	2.20	0.50	450.0	100.0	27.0
	90 min	2.32	2.28	0.50	400.0	100.0	32.0
	120 min	2.17	2.14	0.50	400.0	100.0	24.0
	150 min	2.30	2.27	0.50	400.0	100.0	24.0

a P is short for pectinase, C is short for cellulase.

Table 1 shows that the yield reached 20.0% with pretreatment for 30 min, 27.0% for 60 min, 32.0% for 90 min, and 24.0% and 24.0% for 120 min and 150 min, respectively. It can be concluded that the yield from the pre-treatment time of 90 min is greater as compared to the other four groups. This shows that when the ratio of bio-enzyme is pectinase : cellulase = 1 : 1 for 90 min, this will further accelerate the cleavage of cellulose 1,4 bonds; *i.e.*, the yield of nanocellulose is relatively high under this set of conditions and the hydrolysis efficiency of is greater.

#### 3.1.2 FTIR analysis

**Fig. 3:**
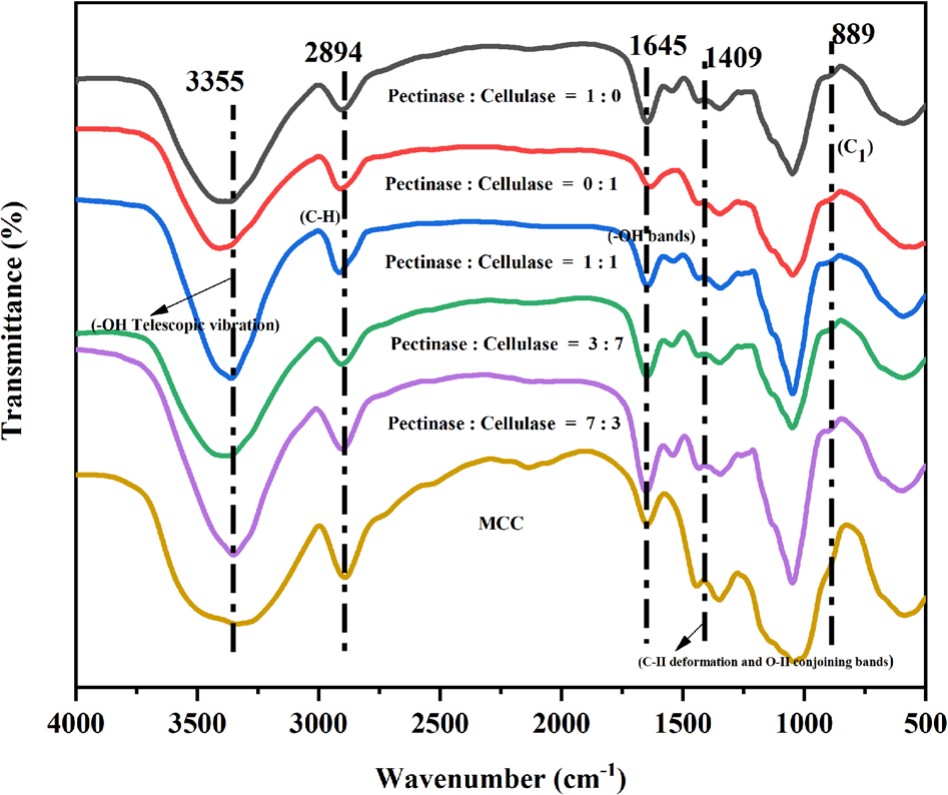
FTIR spectra of MCC and the nanocellulose obtained from different bio-enzyme ratios.

**Fig. 4:**
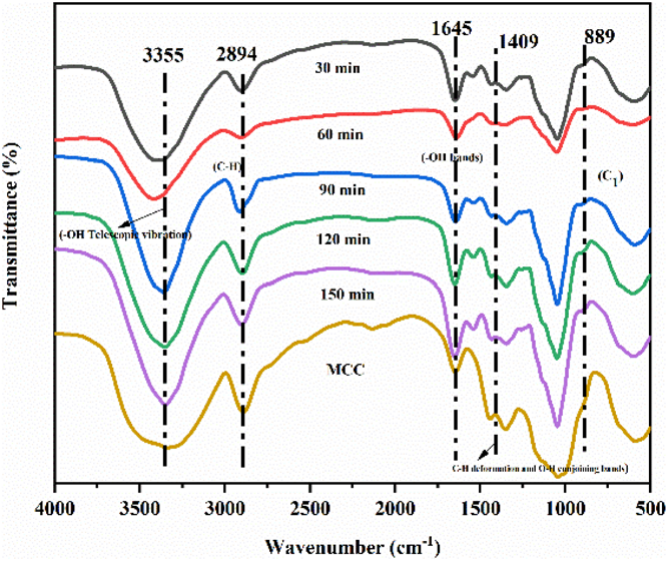
FTIR spectra of MCC obtained after different pretreatment times.

Different bio-enzyme ratios and different bio-enzyme treatment times were investigated using FTIR spectroscopy. FTIR signals near 3355 cm^−1^ (3500–3000 cm^−1^) were attributed to hydrogen bond O–H stretching vibrations and intramolecular and intermolecular hydrogen bond bending vibrations.^34,35^ The absorption band near 2894 cm^−1^ was attributed to C–H stretching vibrations.^36,37^ The peak at 1645 cm^−1^ was associated with the –OH bending of absorbed water.^38,39^ The band at 1409 cm^−1^ was attributed to the C–H deformation (asymmetry) and conjoined O–H bands in cellulose.^40,41^ The isotopic carbon (C_1_) vibrations were observed at 889 cm^−1^.^42,43^ The overall trend of FTIR curves of nanocellulose prepared with different pre-treatment times and different bio-enzyme ratios was consistent. Fig. 3 and 4 show the FTIR spectra of nanocellulose and bio-enzyme-pre-treated MCC, indicating that ultrasonic treatment did not change the chemical structure of the nanocellulose, which maintained the basic structure of natural cellulose. On the other hand, it also indicates that the special characteristics exhibited by nanocellulose were derived from its nano-size effect. This shows that the different ratios of bio-enzymes further accelerated the cleavage of the cellulose-1,4 bond, which was more effective than that of a single bio-enzyme, thus providing the best conditions for forming nanocellulose.

#### 3.1.3 XRD analysis

**Fig. 5:**
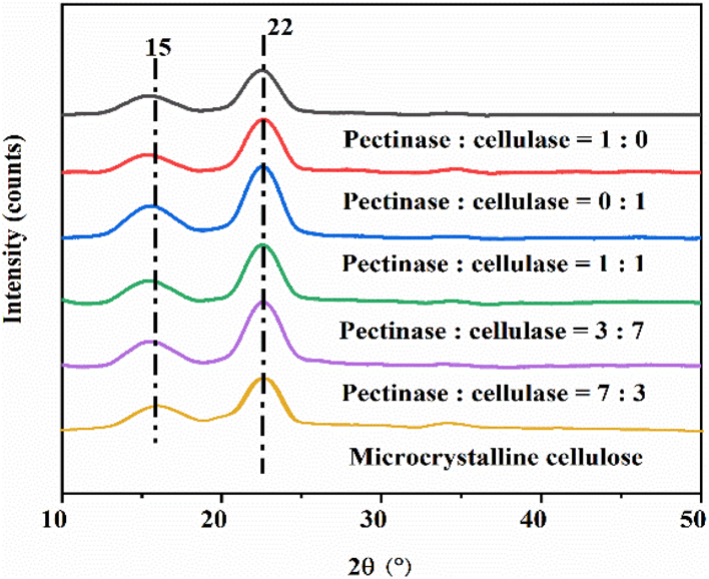
XRD patterns of MCC and the nanocellulose obtained from different bio-enzyme ratios.

**Fig. 6:**
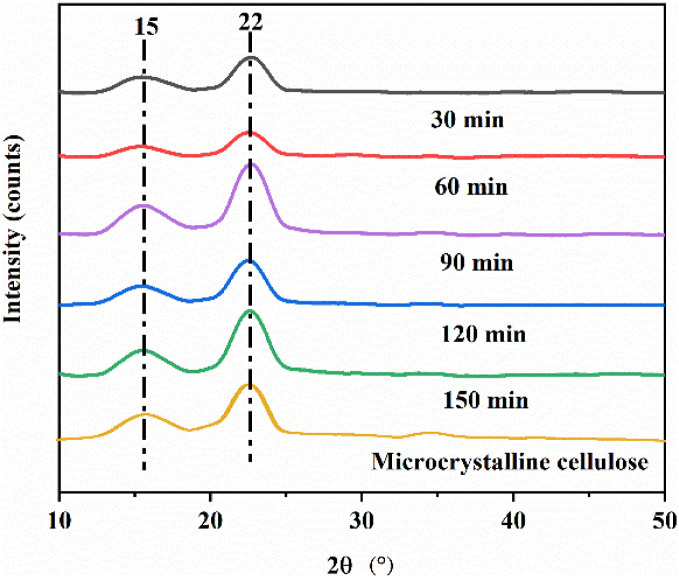
XRD patterns of MCC and the nanocellulose obtained for different pretreatment times.

The XRD patterns of MCC treated with a single bio-enzyme for different times are shown in Fig. 5 and 6. Two diffraction peaks were observed at 2*θ* = 15° and 22° corresponding to the (101) crystal plane and (200) crystal plane. There is only one peak in the (200) crystal plane, therefore, this type belongs to cellulose type I. Bio-enzymes accelerate the breakage of the cellulose-1,4 bond. The breaking of hydrogen bonds and the forming of hydrogen bonds contributed to the formation of the cellulose. The degree of bond breakage can lead to the formation of different nanoscale units with various diameters. The average diameter was reflected in the SEM analysis and it was proven that the nanocellulose that was formed was still natural cellulose.^44–46^ As can be seen in Table 2, MCC was treated by heating and stirring + bio-enzymes + ultrasonic treatment. The crystallinity of nanocellulose obtained by treating MCC with different bio-enzyme ratios for 90 min, was 67.9%, 61.3%, 60.1%, 62.3% and 73.5%, respectively. The crystallinity of nanocellulose obtained by treating MCC with different times for pectinase : cellulase = 1 : 1, was 60.4%, 63.2%, 73.5%, 70.1% and 62.6%, respectively. The highest crystallinity was obtained for MCC treated under the conditions of pectinase : cellulase ratio = 1 : 1, 90 min, at 50 °C.

**Table 2:** The crystallinity of the synthesized nanocellulose

Sample category		CrI (%)
Microcrystalline cellulose		64.0
90 min	P : C = 1 : 0	67.9
	P : C = 0 : 1	61.3
	P : C = 1 : 1	73.5
	P : C = 3 : 7	60.1
	P : C = 7 : 3	62.3
P : C = 1 : 1	30 min	60.4
	60 min	63.2
	90 min	73.5
	120 min	70.1
	150 min	62.6

#### 3.1.4 Summary

Nanocellulose was prepared by treating MCC with a single bio-enzyme and with ratios of bio-enzymes for different pretreatment times. FTIR spectroscopy analysis revealed that there was no change in the chemical structure of cellulose; the basic structure of natural cellulose was maintained. According to XRD analysis, the crystal form of nanocellulose obtained from MCC retained cellulose type I. Under the conditions of pectinase : cellulase = 1 : 1, 90 min, and 50 °C, the crystallinity of nanocellulose was 32.0%.

The unique properties of MCC include natural renewability and biodegradability. The experiment did not involve the use of toxic substances. For MCC treated with bio-enzymes, different proportions of bio-enzymes can catalyze the formation of nanocellulose, and the degree of nanocellulose crystallinity was different. The fracture degree of the uncertain area was also different, resulting in different diameters of nanocellulose. The experiment showed that the highest efficiency of preparing nanocellulose was achieved when MCC was treated with the HSt-P/C-Ultr method, pectinase : cellulase = 1 : 1, 90 min, 50 °C, and the yield of nanocellulose was 32.0%.

### 3.2 SEM analysis

**Fig. 7:**
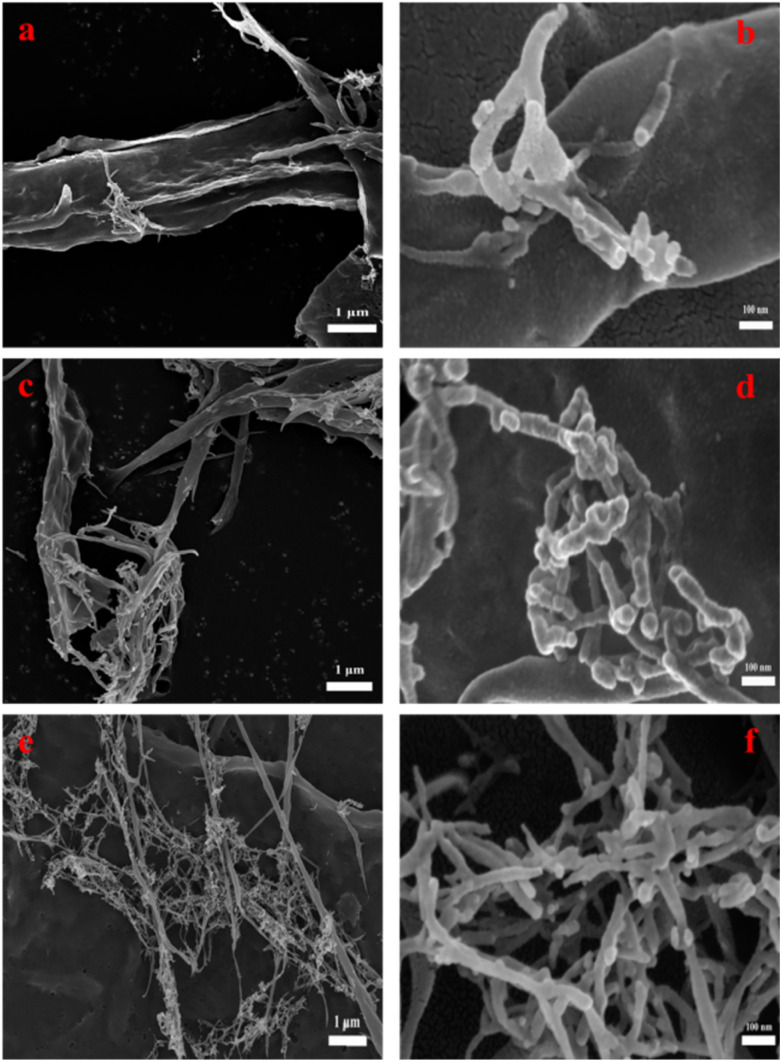
The SEM images of CNF at 30 min (a, b); 90 min (c, d); 120 min (e, f).

**Fig. 8:**
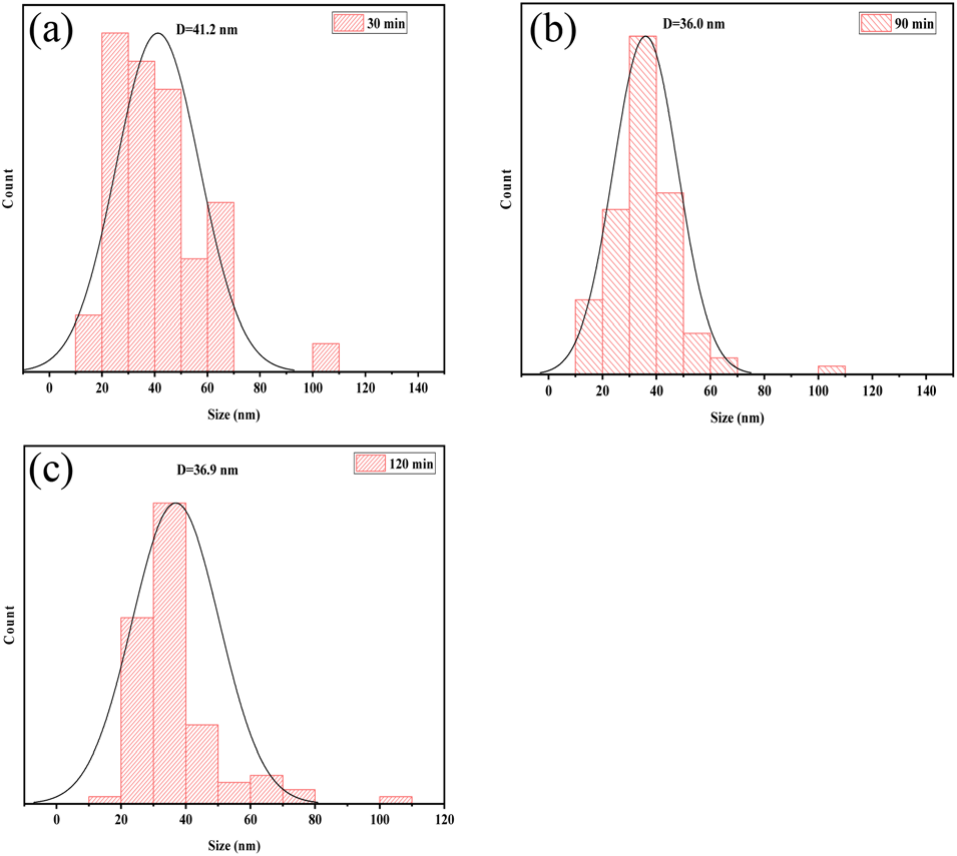
The size of CNF at 30 min (a), 90 min (b), 120 min (c).

The shape of the cellulose material after bio-enzyme pretreatment was analyzed using SEM as shown in Fig. 7. The microscopic morphology of nanocellulose was a mesh-like structure, and the nanocellulose fiber (CNF) was obtained by this method. As shown in Fig. 8, by combining Image J and Origin software, it was possible to calculate the average width of the CNF. This was determined to be 36.0 nm from 90 min of bio-enzyme pretreatment, and the average diameters of CNF obtained from 30 min and 120 min treatment were 41.2 nm and 36.9 nm, respectively. This is attributed to the partial degradation of the amorphous region of cellulose. During pretreatment with bio-enzymes, it was shown that the average diameter of CNF decreased with increased pre-treatment time. At 120 min, the average diameter of CNF did not continue to decrease, which indicates that the increase in reaction time led to the excessive hydrolysis of nanocellulose, which is attributed to the swelling effect of cellulose. Therefore, the bio-enzyme ratio of pectinase : cellulase = 1 : 1 for 90 min was optimal for the bio-enzyme pretreatment of CNF.

### 3.3 TEM analysis

**Fig. 9:**
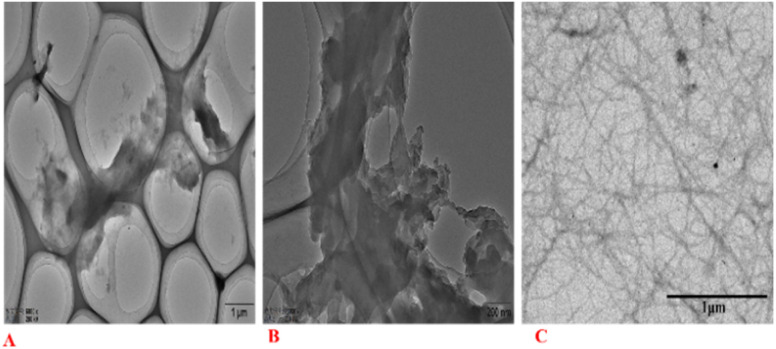
TEM images of CNF for 90 min, P : C = 1 : 1 (A–C).

TEM images of nanocellulose after bio-enzyme hydrolysis (90 min, 50 °C) with ultrasonic treatment are shown in Fig. 9. The internal structure of cellulose still shows the agglomeration phenomenon.^47^Fig. 9 shows nanoscale widths between 20 and 50 nm. The nanofibers were also entangled in a network structure of thin filaments, caused by the interaction between hydroxyl groups.^48,49^ This greatly increased the interface with the polymer bonding region and had a toughening effect, showing the full nano-effect in the polymer. The network structure of the filaments of the nanocellulose fibers is shown in Fig. 9C. Therefore, the nanocellulose prepared in this study is CNF. From Fig. 9, we can also see the network structure of nanocellulose and the filament structure of nanocellulose, which can explain the use of bio-enzymes to accelerate the breaking of cellulose-1,4 bonds, and study the effects of the ratios of bio-enzymes and pretreatment time on the properties of nanocellulose. We can also see that the CNF dispersion was relatively uniform. The CNF dispersion in water was in a colloidal state and relatively stable. The concentration of CNF prepared in this experiment was low, and as the CNF dispersion concentration was 0.10%, it was in a relatively stable suspension.

### 3.4 TG analysis

**Fig. 10:**
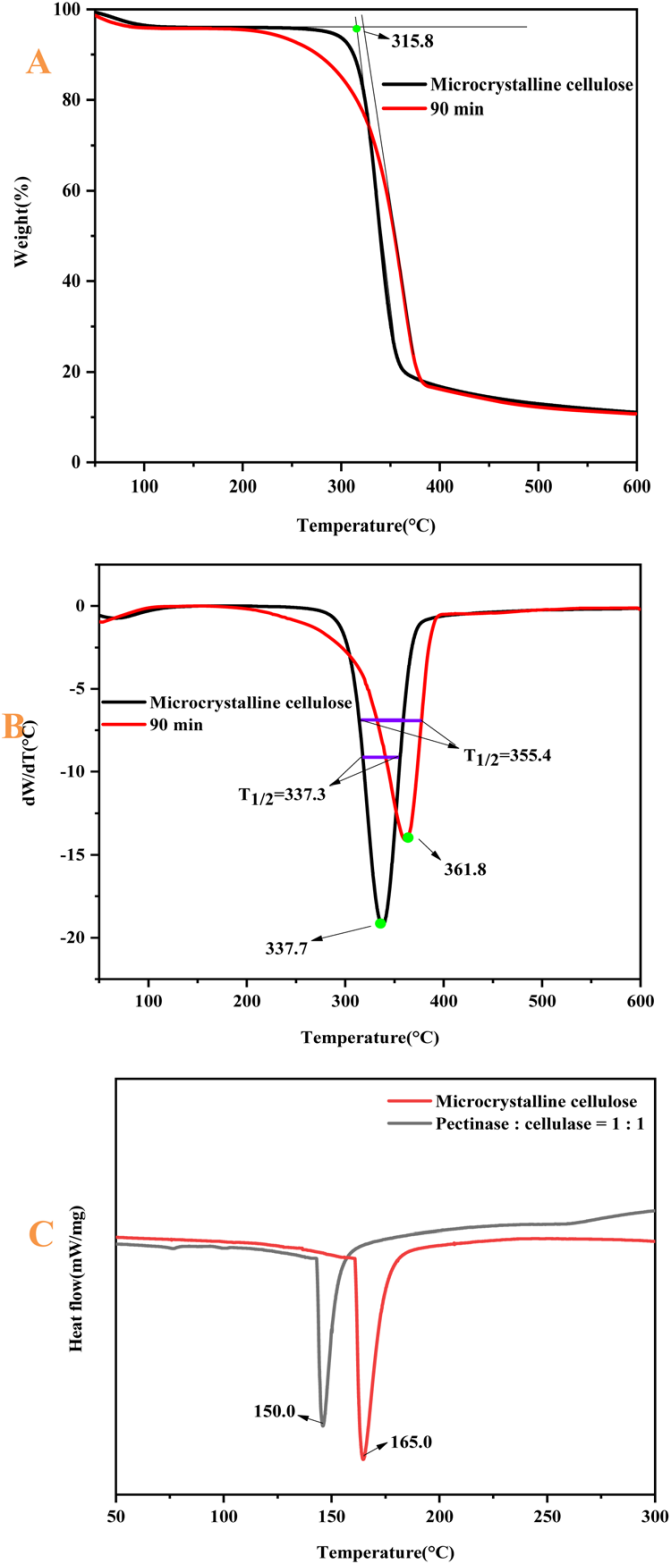
Mass loss images of MCC and nanocellulose (A); DTG images of MCC and nanocellulose (B); DSC images of MCC and nanocellulose (C).

The thermogravimetric curves of MCC and CNF are shown in Fig. 10. The thermal decomposition of cellulose includes three processes: dehydration of cellulose molecular chains (33–140 °C), depolymerization (140–240 °C) and decomposition of glucosyl units (240–430 °C),^50^ and the mass loss of MCC. The CNF in the initial stage (33–140 °C) was mainly due to the volatilization of free water adsorbed on the fiber surface. The starting thermal decomposition temperature of MCC was 315.8 °C. The rate of thermal decomposition was maximum at 337.7 °C.^51,52^ After that, it entered the slow decomposition stage, and the mass residual rate of the sample at 599.7 °C was 11.1%. The starting thermal decomposition temperature of CNF was 322.7 °C. The temperature at which the maximum thermal decomposition rate was reached was 361.8 °C. The mass residual rate of the sample at 599.7 °C was 10.7%.^53^ The maximum thermal decomposition rate of CNF was 24.1%, which was higher than that of MCC, with a small difference of 0.40%. Compared with MCC, the TG and DTG curves of CNF were shifted toward high temperatures, indicating that the thermal stability of the prepared CNF was enhanced. This paper shows that under the conditions of pectinase : cellulase = 1 : 1, 90 min, and 50 °C, the best efficiency of the bio-enzyme was exerted. Therefore, the thermal stability of the nanocellulose was observed from the thermogravimetric analysis. The reaction conditions of the preparation method were mild, and the damage to the crystalline region of cellulose was small. After the amorphous region was hydrolyzed, the ordered crystalline region was retained. The order of the cellulose macromolecule arrangement was enhanced by ultrasonic treatment and thus, the crystalline region of the prepared CNF was arranged, so the thermal stability was enhanced.

The individual temperatures for the thermal degradation of each sample are listed using Table 3. *T*_0_ is the initial degradation temperature of the sample, *T*_1/2_ is the temperature corresponding to 50% weight loss of the sample, and *T*_max_ is the temperature corresponding to the highest thermal degradation peak. The DSC analysis of pectinase : cellulase = 1 : 1 and MCC was carried out as shown in Fig. 10C. The curing temperature of pectinase : cellulase = 1 : 1 is lower than that of MCC; *i.e.*, the energy consumption of pectinase : cellulase = 1 : 1 is relatively low. Therefore, the bio-enzyme method of preparing nanocellulose consumes little energy. The data in the table show the characteristic degradation temperature of the nanocellulose samples, indicating that the thermal stability of the nanocellulose fibers was improved.

**Table 3:** Thermal degradation data of MCC and nanocellulose

Sample category	*T*_0_ (°C)	*T*_max_ (°C)	*T*_1/2_ (°C)
Microcrystalline cellulose	315.8	337.7	337.3
P : C = 1 : 1; 90 min	322.7	361.8	355.4

## 4 Conclusion

Microcrystalline cellulose (MCC) was pretreated under the conditions of HSt – P/C – Ultr to prepare nanocellulose. The effects of the ratios of pectinase and cellulase, and the times of bio-enzymes on the yield of nanocellulose were investigated. In the first stage of the experiment, pectinase : cellulase = 1 : 1 was found to be the best ratio of bio-enzymes. The second stage of the experiment indicated that the best hydrolysis time was 90 min.

The yield of nanocellulose obtained was about 32.0%, which was greater than the yield of a single bio-enzyme and other hydrolysis times, indicating the synergistic effects of pectinase and cellulase on the formation of nanocellulose. During the preparation process, it remained as cellulose I. The crystallinity was 73.5%, which was 9.50% higher than that of MCC. It was determined that the nanocellulose fiber (CNF) had a high aspect ratio and a width between 20-50 nm. The nanocellulose prepared under HSt – P/C – Ultr conditions not only conforms to the concept of a green environment but is also an ideal toughening material, which is important for the manufacture of composite materials.

## Author contributions

Conceptualization, X. Wu and L. Zhang; methodology, X. Wu, X. Li and L. Zhang; software, X. Wu; validation, X. Wu, H. Guo, X. Li and L. Zhang; formal analysis, X. Wu, J. Zhao and X. Yuan; investigation, X. Wu, J. Zhao and X. Yuan; writing—original draft preparation, X. Wu, J. Zhao and X. Yuan; writing—review and editing, L. Zhang; visualization, X. Wu and L. Zhang; supervision, D. Ji, and W. Yao; project administration, X. Li and L. Zhang; funding acquisition, X. Li and L. Zhang. All authors have read and agreed to the published version of the manuscript.

## Data availability statement

The data presented in this study are available on request from the corresponding author.

## Conflicts of interest

The authors declare that the research was conducted in the absence of any commercial or financial relationships that could be construed as a potential conflict of interest. There is no conflict to declare.

## Supplementary Material
